# Supplementary material for: Integrated omics dissection of proteome dynamics during cardiac remodeling
Source: Nat Commun. 2018 Jan 9;9:120. doi: 10.1038/s41467-017-02467-3 (PMC5760723; doi:10.1038/s41467-017-02467-3)
Supplement: Supplementary file 1 — Supplementary Information [file 41467_2017_2467_MOESM1_ESM.pdf]

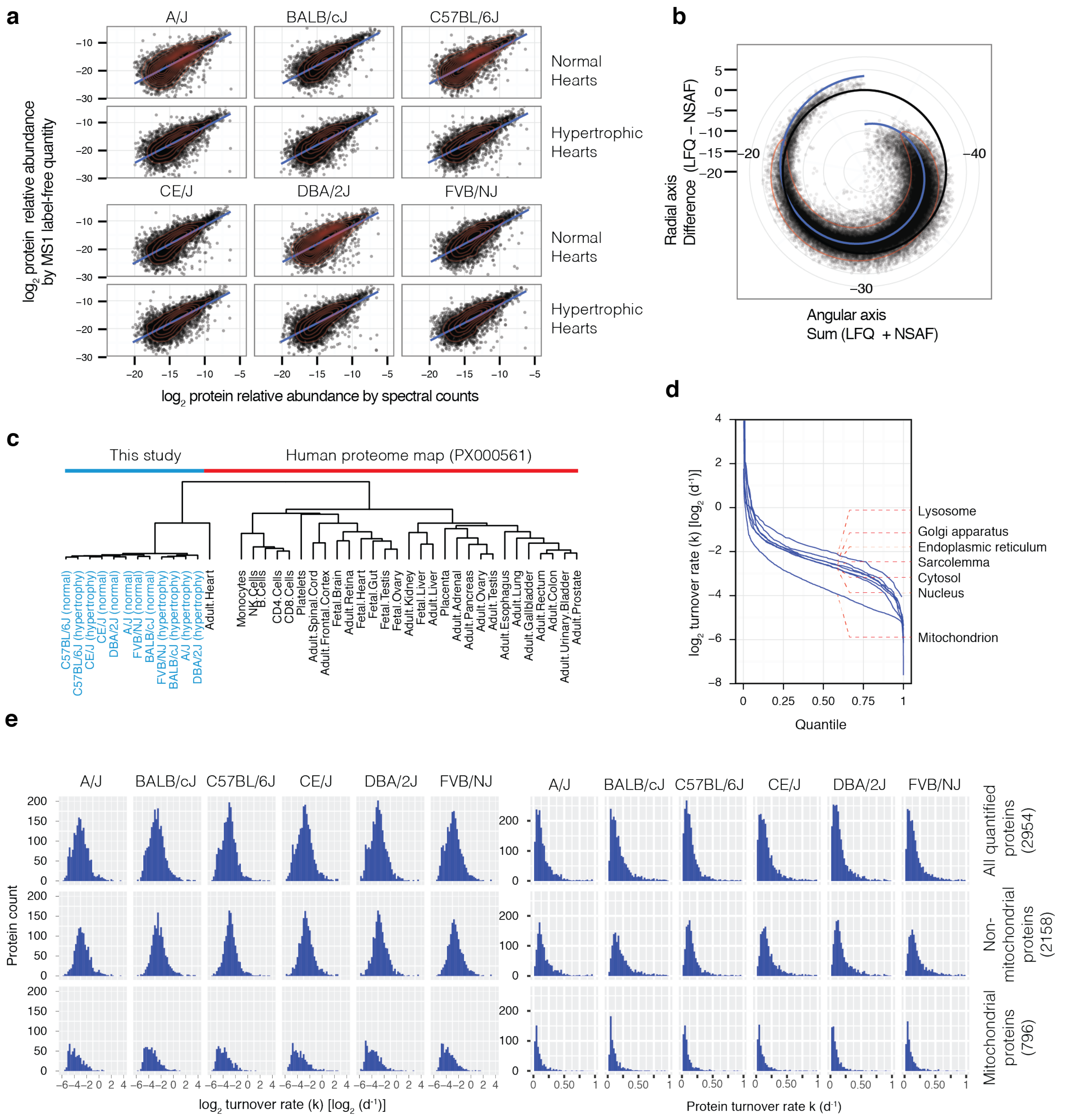


**Supplementary Figure 1. Large-scale method to measure protein abundance and turnover in vivo.** **a:** Distribution of log relative abundance of analyzed proteins as measured by normalized spectral abundance factor (NSAF; x-axis) and chromatographic intensity-based label-free quantification (LFQ; y-axis). **b:** Polar Tukey mean-difference plot showing similar measured values between spectral counts and chromatographic intensity values. **c:** Dendrogram of agglomerative hierarchical clustering of relative protein abundance between experiments in this study and public datasets on human tissues

(PX000561). The relative protein abundance data here are closer to the protein expression profile of the adult mammalian heart than those of any other mammalian tissues examined, demonstrating the specificity of the label free quantification data. **d:** Distribution of log turnover rates (k) (y-axis) of all confidently quantified proteins in all six mouse strains at baseline plotted against their quantiles (x-axis) within each category. The data are separated by the annotated subcellular localizations of the proteins. Many cellular compartments show similar protein turnover distributions, but mitochondrial proteins show distinctly slower turnover than proteins in other compartments. **e:** Histogram of the distribution of $\log_2$ turnover rates ($\log_2$ k; left) and linear turnover rates (k; right) of all proteins quantified in each of the six mouse strains, separated into mitochondrial and non-mitochondrial proteins. The wide distributions of turnover rates in each sample are apparent, as are the significantly lower turnover of mitochondrial proteins vs. non-mitochondrial proteins. Numbers in parentheses denote numbers of proteins quantified in each category.

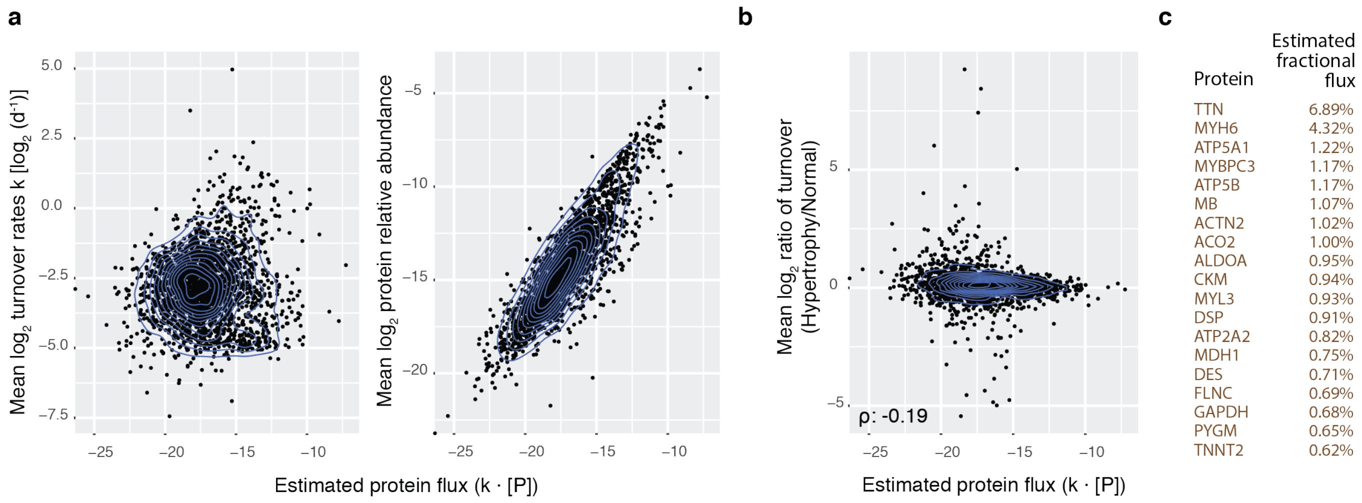


**Supplementary Figure 2. Estimation of protein turnover flux in the dataset.** The average baseline turnover flux of proteins in control hearts of six strains of mice (A/J, BALB/cJ, C57BL/6J, CE/J, DBA2/J, FVB/NJ) is estimated according to Hammond et al. [23]. **a:** Scatter plot of protein turnover flux against protein turnover rate k ($d^{-1}$) (left) and protein abundance (right). Data point: protein species. Protein abundance span a larger dynamic range than turnover and exerts a larger effect on the overall protein turnover flux in the heart. **b:** Scatter plot of baseline turnover flux against $\log_2$ ratio of turnover in hypertrophic vs. normal mouse heart (Spearman's rho = -0.19). **c:** List of 20 myocardial proteins with highest flux in the dataset, after removing likely blood protein contaminants.

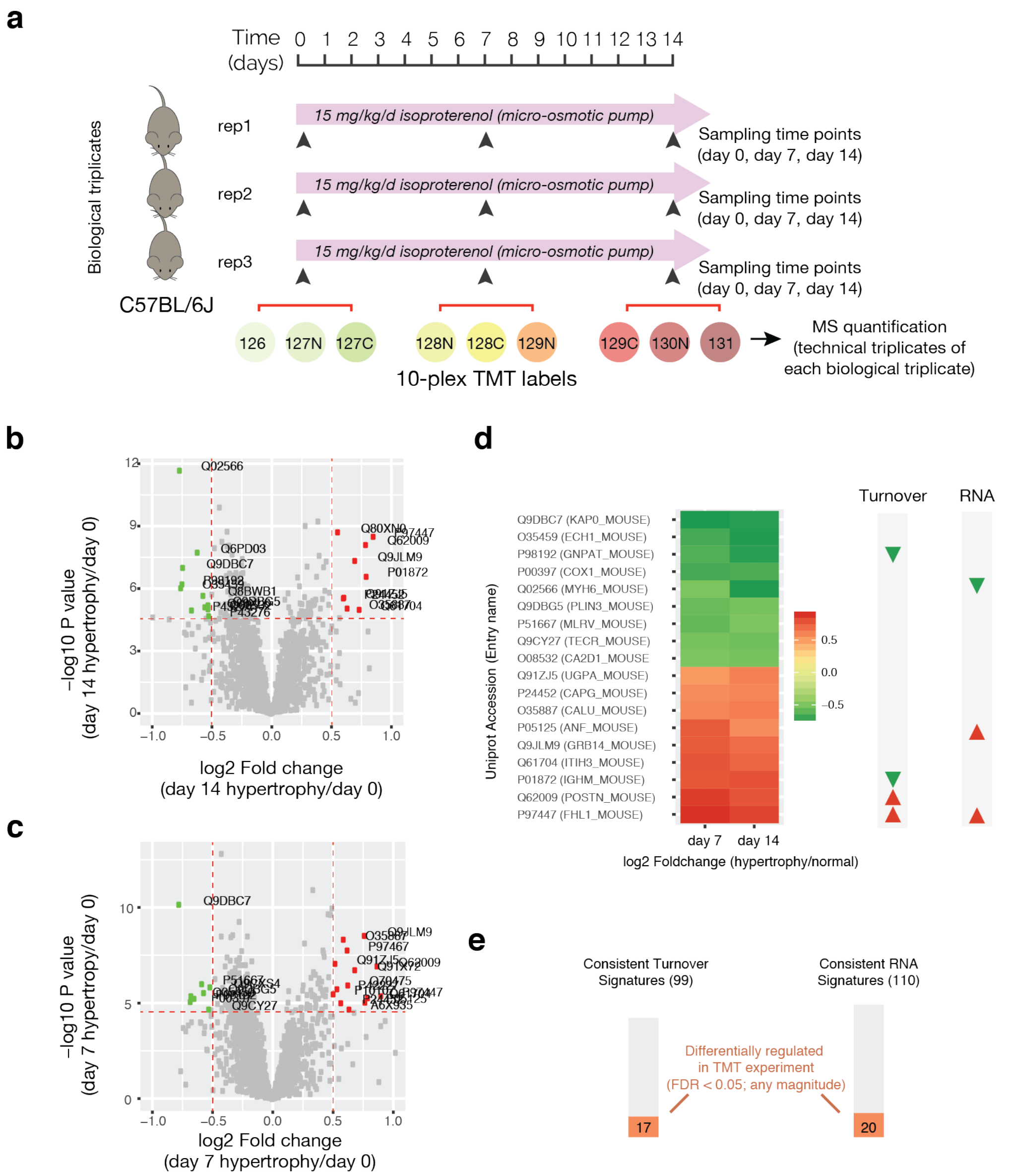


**Supplementary Figure 3.** Protein quantification in hypertrophic mouse hearts using tandem mass tags. **a:** Schematic of tandem mass tag (TMT) experiments. Triplicate groups of C57BL/6J mice were administered with 15 mg·kg$^{-1}$·d$^{-1}$ isoproterenol via microosmotic pumps for up to 14 days. Cardiac proteins were collected at day 0, day 7, day 14 and labeled with 10-plex TMT labels. Each set of TMT-labeled samples (9 channels) were analyzed in technical triplicates. **b-c:** Volcano plots of $-\log_{10}$ P value over $\log_2$ fold change in (B) day 14 of hypertrophy and (C) day 7 of hypertrophy against control sample. **d:** Heat map of differentially regulated proteins in TMT experiments at FDR < 0.05 and absolute $\log_2$ fold-change of 0.5. Right: Only four of the differentially regulated samples were also consistent turnover signatures; only three were consistent RNA signatures. **e:** Upon relaxing the TMT signature criteria (FDR < 0.05; any magnitude), only 17 of 99 turnover signatures and 20 out of 110 RNA signatures show

differential regulation at the protein abundance level. Hence taken together the data do not support that an orthogonal method of protein quantification is able to recapitulate turnover and RNA signatures.

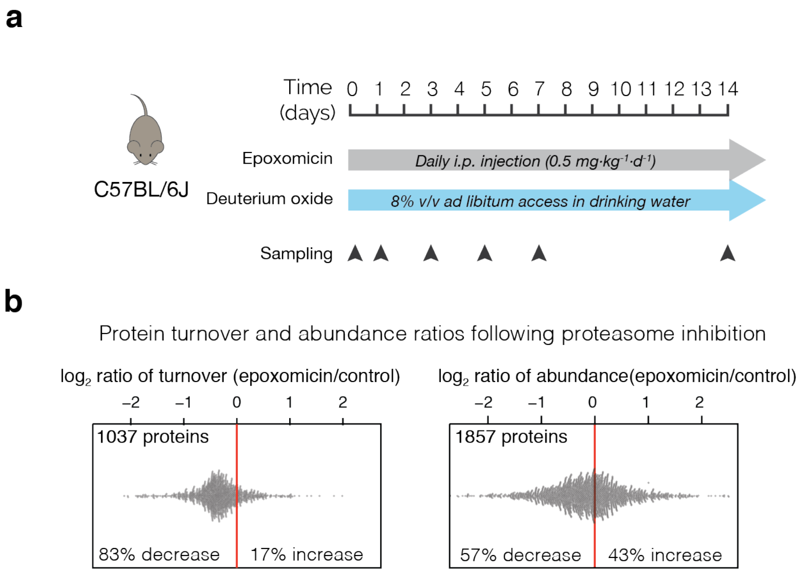


**Supplementary Figure 4.** Protein turnover rates are influenced by proteasomal degradation. **a:** Schematic of experiment. Groups of C57BL/6J mice were administered 0.5 mg·kg$^{-1}$·d$^{-1}$ epoxomicin via daily intraperitoneal injection for up to 14 days, concomitant to in vivo deuterium labeling to examine the influence of proteasomal inhibition on protein turnover rates. **b:** 83% of measured proteins showed decreased turnover rates under epoxomicin treatment (1,037 proteins compared). By contrast, 57% of compared proteins had decreased abundance.

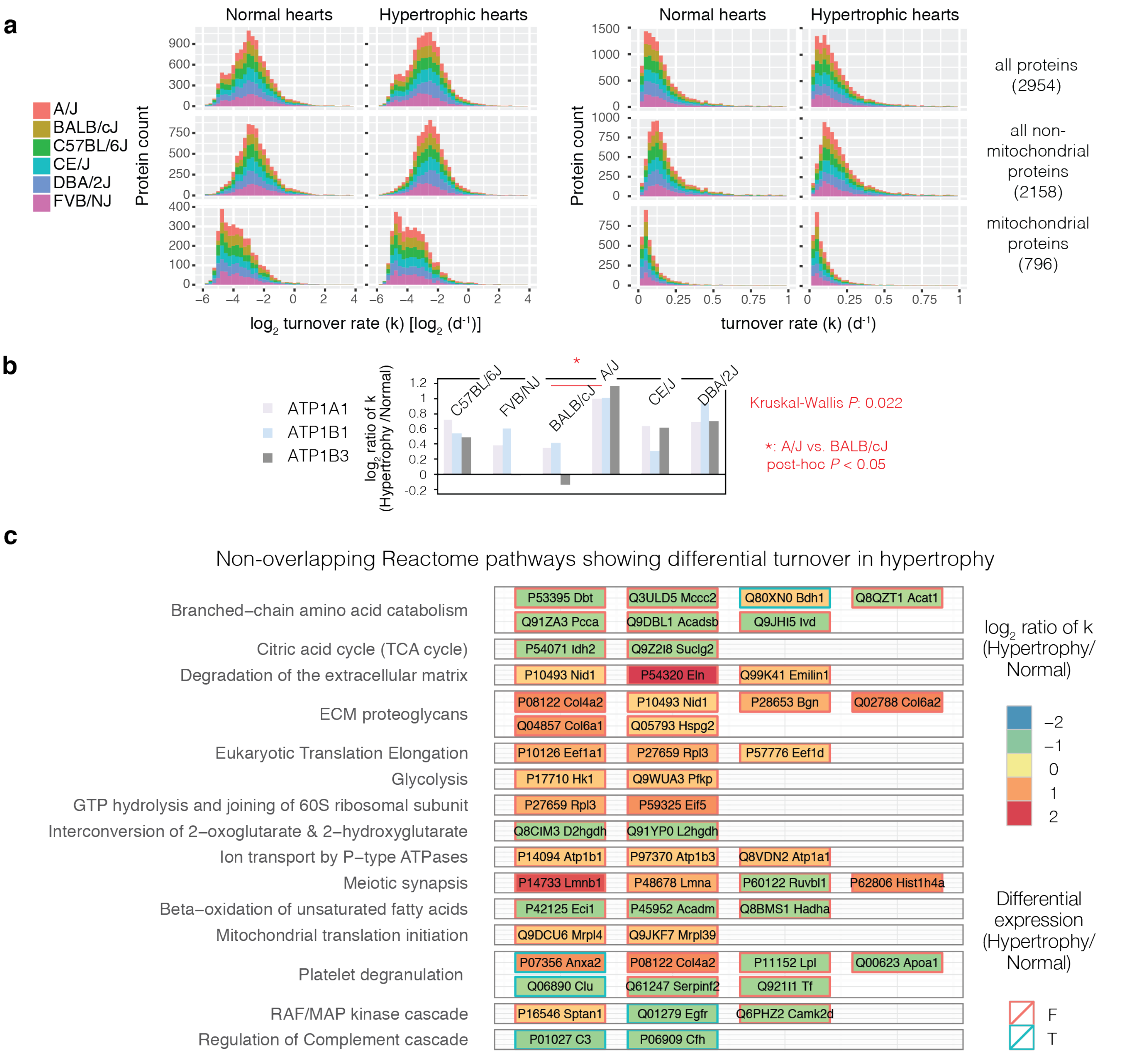


**Supplementary Figure 5**. **Comparison of protein turnover in normal and hypertrophic heart. a:** The distributions of $\log_2$ turnover rates ($\log_2$ k; left) (x-axis) and linear turnover rates (k; right) in normal and hypertrophy hearts are shown. Histograms from each of the analyzed mouse strains (marked by colors) are shown. **b:** Strain-specific differences in turnover rate ratio of $Na^+/K^+$ ATPases ATP1A1/ ATP1B1/ ATP1B3 hypertrophy over normal hearts. Significant differences were observed between the ratios of $Na^+/K^+$ ATPase in hypertrophic vs. normal hearts in the six mouse strains (Kruskal-Wallis *P*: 0.022; post-hoc $P < 0.05$ between A/J and BALB/cJ). **c:** Non-overlapping Reactome pathways enriched in proteins showing differential turnover in cardiac hypertrophy. Color fills on the individual protein blocks denote $\log_2$ ratio of protein turnover in hypertrophic vs. normal hearts as shown in the legends. Borders denote whether the proteins also showed substantial differential expression on the same model. The data show

a number of critical cardiac pathways and components with substantial changes in protein turnover dynamics, including glycolysis, TCA cycle, and extracellular matrix proteins, which are not always accompanied by abundance changes.

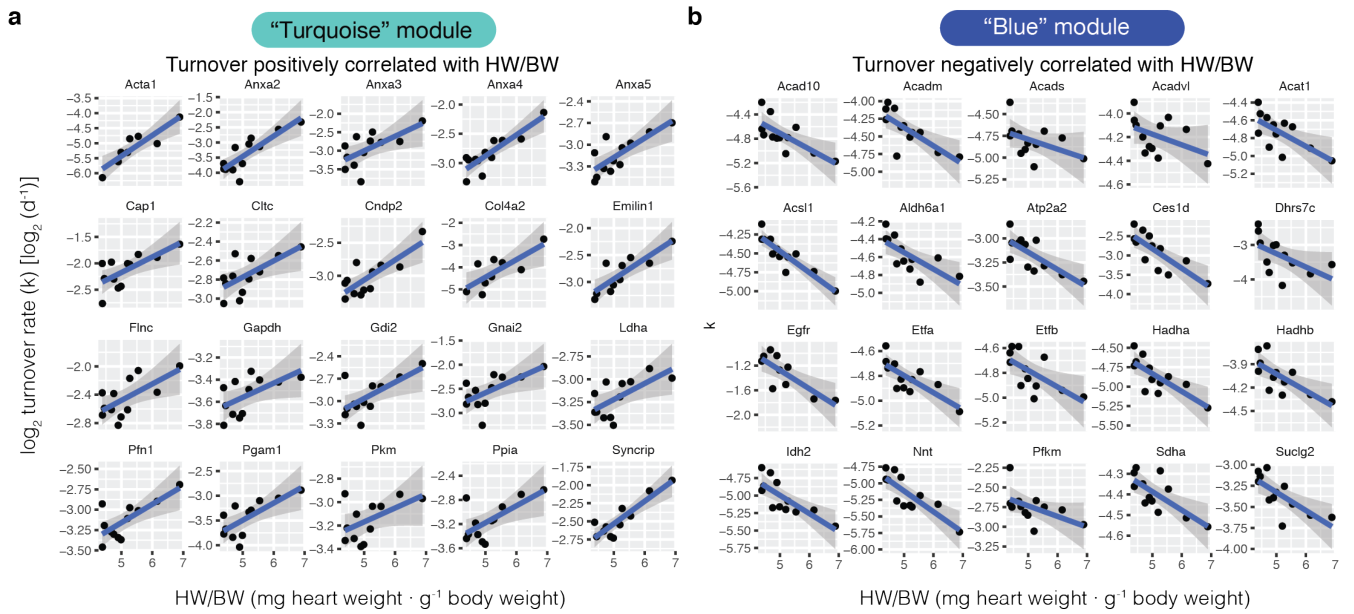


**Supplementary Figure 6. Correlation of protein turnover clusters with cardiac hypertrophy. a:** Turnover of proteins in the module labeled "Turquoise" is positively correlated with hypertrophy, as in Figure 5C. Data are presented in logarithmic scales here. **b:** Turnover of proteins in the "Blue" module is negatively correlated with hypertrophy, as in Figure 5D. Data are presented in logarithmic scales here.

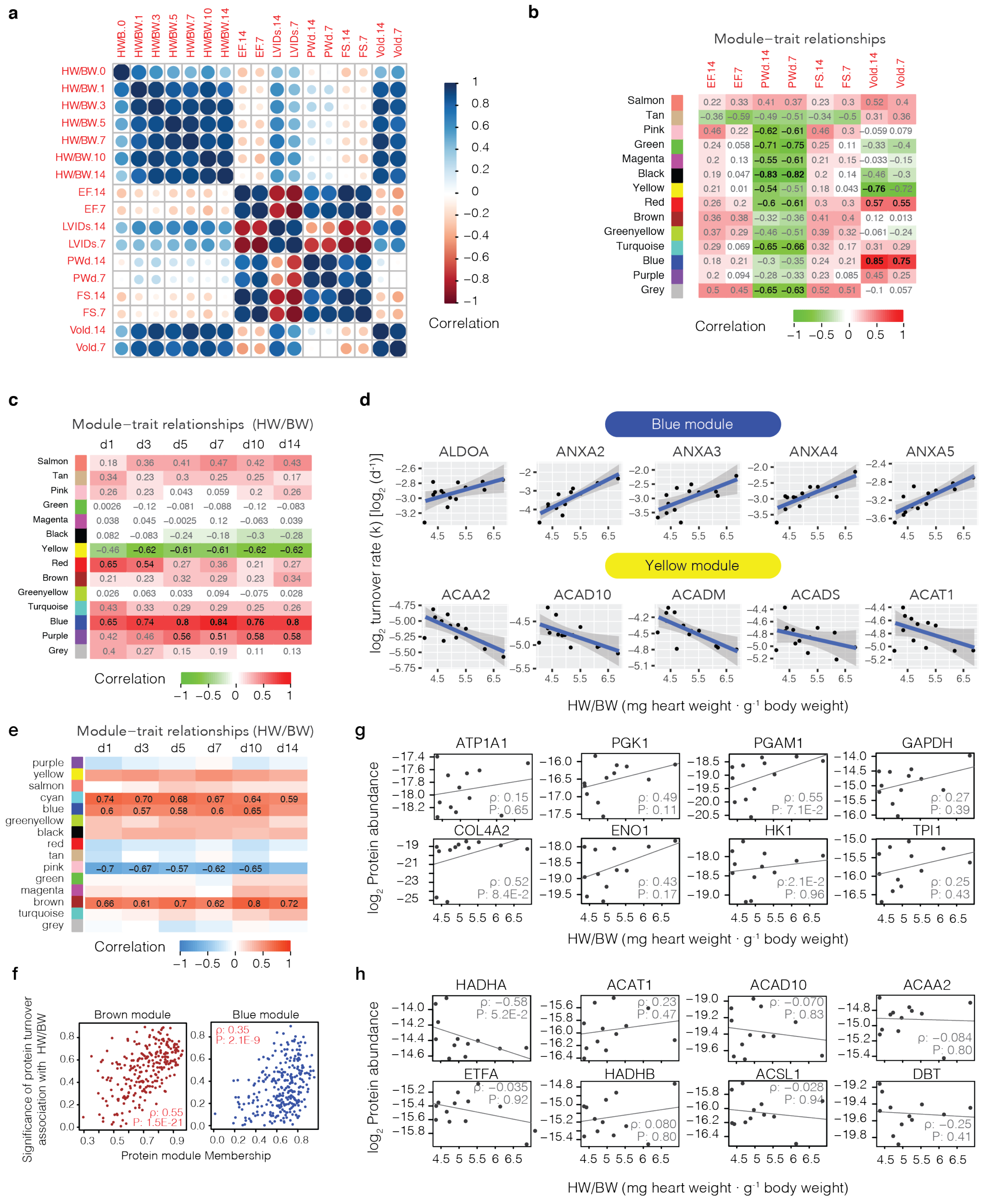


**Supplementary Figure 7. Correlation of protein turnover to cardiac traits from additional datasets.**

**a:** Comparison of heart-weight-to-body-weight ratio (HW/BW) data at multiple time points (0, 1, 3, 5, 7,

10, 14 days) in this study to echocardiogram heart traits from an independent cohort of mice of the same strains under isoproterenol stimulation. Size and color of circles denote strength and directionality of pairwise correlation between two traits. The measured HW/BW data in this study are strongly and positively correlated with left ventricular internal diameter (LVID) and end diastolic volume (Vold). **b:** Correlation between protein turnover and selected external echocardiographic traits (ejection fraction EF, fractional shortening FS, left ventricular diastolic posterior wall thickness PWd, and Vold). The protein modules above were significantly and positively correlated with Vold at day 7 and day 14 of isoproterenol treatment. Heat map colors and numbers denote correlation coefficient. Correlation coefficients in black are suggestive ($P$<0.05); correlation coefficients in black and bold are significant (adjusted $P$<0.05). An additional module (black) shows significant correlation with PWd at day 7 and day 14, but correlations between individual black module proteins and the trait are not significant following multiple testing correction. **c:** Clustering of protein turnover against HW/BW data following the inclusion of an additional mouse strain (Hsd:ICR CD-1) from a published study [21]. Network structures and protein-trait correlations are preserved. Heat map colors and numbers denote correlation coefficient. Correlation coefficients in black are suggestive ($P$<0.05); correlation coefficients in black and bold are significant (adjusted $P$<0.05). **d:** Example protein-trait relationships from data with seven mouse strains. Abscissae: HW/BW (mg per g); ordinates: $\log_2 k$. **e:** An independent clustering analysis was performed using the relative expression levels of cardiac proteins. Heat map shows the strength of positive (red) or negative (blue) correlations between protein abundance in a module with heart weight at multiple time points. No significant correlations remained after multiple-testing correction. The correlation coefficients of all suggestive ($P$<0.05, $P_{adj}$>0.05) module-trait correlations are printed in gray. **f:** The most strongly associated (brown) module contained 277 proteins, which were suggestively correlated with heart weight ($r$: 0.81, $P$: 0.001, $P_{adj}$: 0.15), and which were significantly enriched in ER ($P_{adj}$: 0.035) and plasma membrane ($P_{adj}$: 0.012) proteins. Abundance ratios of selected proteins from the (**g**) blue and (**h**) yellow modules in the clustering analysis of turnover data show insignificant correlations between $\log_2$ protein abundance (y-axis) and HW/BW (x-axis). An exception is HADHA, whose abundance is negatively correlated with heart weight ($P$: 0.052).

| HW/BW (mg/g) | C57BL/6J | FVB/NJ | BALB/CJ | A/J | CE/J | DBA/2J |
|---|---|---|---|---|---|---|
| Day 0 average | 4.34 | 4.45 | 4.46 | 4.35 | 4.53 | 5.37 |
| Day 0 s.d. | 0.24 | 0.22 | 0.29 | 0.26 | 0.17 | 0.27 |
| Day 1 average | 5.35 | 5.23 | 5.18 | 4.95 | 4.8 | 6.25 |
| Day 1 s.d. | 0.24 | 0.22 | 0.24 | 0.22 | 0.26 | 0.27 |
| Day 3 average | 5.79 | 5.8 | 5.82 | 5.75 | 5.38 | 7.58 |
| Day 3 s.d. | 0.24 | 0 | 0.34 | 0.29 | 0.29 | 0.62 |
| Day 5 average | 5.75 | 5.04 | 6.06 | 6.13 | 5.49 | 7.02 |
| Day 5 s.d. | 0.09 | 0.45 | 0.26 | 0.38 | 0.47 | 0.4 |
| Day 7 average | 5.6 | 4.94 | 5.83 | 6.03 | 5.55 | 6.94 |
| Day 7 s.d. | 0.41 | 0.07 | 0.21 | 0.26 | 0.37 | 0.65 |
| Day 10 average | 4.84 | 5.1 | 5.82 | 5.32 | 5 | 6.97 |
| Day 10 s.d. | 0.25 | 0 | 0.27 | 0.37 | 0.21 | 0.13 |
| Day 14 average | 4.78 | 5.2 | 6.16 | 5.55 | 5.27 | 6.89 |
| Day 14 s.d. | 0.31 | 0 | 0.24 | 0.55 | 0.13 | 0.62 |
| BW (g) | | | | | | |
| average | 21.66 | 25.67 | 27.78 | 26.75 | 23.63 | 29.6 |
| s.d. | 2.11 | 3.27 | 2.2 | 2.25 | 2.53 | 2.18 |

**Supplementary Table 1.** Heart-weight-over-body-weight ratios (HW/BW) in six inbred strains of mice following up to 14 days of isoproterenol administration are shown. s.d.: standard deviation. The six inbred strains commonly used in biomedical research were selected from a panel of 100+ inbred and recombinant inbred strains for their varying responses to isoproterenol.
