# Supplementary material for: Integrated omics dissection of proteome dynamics during cardiac remodeling
Source: Nat Commun. 2018 Jan 9;9:120. doi: 10.1038/s41467-017-02467-3 (PMC5760723; doi:10.1038/s41467-017-02467-3)
Supplement: Supplementary file 3 — Description of Additional Supplementary Files [file 41467_2017_2467_MOESM3_ESM.pdf]

Description of Additional Supplementary Files

File Name: Supplementary Data 1
Description: List of Discovered Disease Signatures. Proteins with consistently altered turnover, protein abundance, or transcript abundance in hypertrophic vs. normal hearts are tabulated. Uniprot = Uniprot accession. The Uniprot accession of the 70 disease signatures nominated via turnover only are bolded. GN = Gene name. PN = Protein name. rna_abundance = Shows consistently altered transcript abundance in hypertrophy among the genetic backgrounds examined. protein abundance = Shows consistently altered protein abundance in hypertrophy among the genetic backgrounds examined. turnover = Shows consistently altered protein turnover in hypertrophy among the genetic backgrounds examined. HL = Mean basal half-life of proteins among the genetic backgrounds examined. D = Mean log2 turnover ratio of proteins in hypertrophy among the genetic backgrounds examined. Flux = Mean log2 protein turnover flux among the genetic backgrounds examined.

File Name: Supplementary Data 2
Description: Reactome Pathway Groups Implicated in Hypertrophy via all 273 Implicated Disease Proteins. Known interactions (from IntAct, Mint, and BioGrid) between the 70 disease proteins implicated via turnover and the remainder of disease proteins. The 13 Reactome pathways highlighted in red are implicated only following the addition of turnover in our dataset. Group = All Reactome pathway terms within the pathway group. Rep = Representative Reactome pathway term within the group. Name = Name of the representative Reactome pathway term within the group. N = Number of disease proteins that are member of the Pathway group.

File Name: Supplementary Data 3
Description: Known Protein-Protein Interactions of Disease Proteins Discovered via Turnover. Known interactions (from IntAct, Mint, and BioGrid) between the 70 disease proteins implicated via turnover and the remaining disease proteins are shown. Columns: int.mouse.A = Uniprot accession of first interactor. GN.A = Gene name of first interactor. int.mouse.B = Uniprot accession of second interactor. GN.B = Gene name of second interactor.

File Name: Supplementary Data 4
Description: Meta-analysis of Transcript and Protein Expression of Disease Signatures.
For each of the 273 candidate disease proteins for cardiac hypertrophy, this table lists the comparison of their corresponding normalized ratios in protein expression and turnover fromt 29 transcriptomics datasets retrieved from NCBI GEO. Uniprot = Uniprot accession. dataset_# = Dataset number corresponding to the index in Figure 3.

File Name: Supplementary Data 5

Description: Turnover Rates of Protein-Protein Interaction Pairs. This table lists 7767 non-self-interaction pairs from BioGrid and their average measured turnover rates in the hearts of the strains of mice in which they are quantified. int.mouse.A = Uniprot accession of first interactor; int.mouse.B = Uniprot accession of second interactor; avg.k1 = Mean turnover rate constant of the first interactor among strains; avg.k2 = Mean turnover rate constant of the second interactor among strains; diff = Difference in mean turnover rate constants between the interactors.
